# Evaluation of depth of cure and knoop hardness in a dental composite, photo-activated using different methods

**DOI:** 10.4103/0972-0707.44055

**Published:** 2008

**Authors:** Mithra N Hegde, Priyadarshini Hegde, Babita Malhan

**Affiliations:** Department of Conservative Dentistry and Endodontics, A. B. Shetty Memorial Institute of Dental Sciences, Deralakatte, Mangalore, Karnataka, India

**Keywords:** Depth of cure, knoop hardness, photo-activation methods

## Abstract

The study aimed at evaluating the depth of cure and knoop hardness of a microfine-hybrid composite resin that was photo-activated using different methods. A bipartite brass mold was filled with composite resin and photo-activation was performed using four methods: (1) Intermittent method using quartz tungsten halogen (QTH) light curing unit (LCU) (2) Continuous method (QTH) (3) Exponential method (QTH) (4) Continuous method using light-emitting diode (LED). Depth of cure was measured at the unexposed bottom surface of the specimen using microtester as a penetrometer. The surfaces exposed to light were subjected to knoop hardness testing, using a digital microhardness tester. Knoop hardness measurements were obtained at the top surface and at depths of 1, 2, 3, 4 and 5 mm. The data was analyzed using anova and Tukey's test (5%). Results showed that the depth of cure was higher with the intermittent method (QTH), followed by the continuous method (QTH), the exponential method and the continuous method (LED). At the top surface and up to 1 mm, continuous method (LED) demonstrated the highest knoop hardness number (KHN). At 2 mm and up to 5 mm, intermittent method (QTH) presented the highest KHN and continuous method (LED) showed the lowest KHN. At all depths, continuous method (QTH) showed higher KHN, as compared to the exponential method (QTH), except at 2 mm where both showed no significant difference.

## INTRODUCTION

Light-activated composite resin restoratives have been widely applied in clinical dentistry, since their introduction in the 1970's, when significant changes with satisfactory application both in anterior and posterior teeth became possible. However, characteristics such as composition, light intensity and exposure time can modify the final properties of the material and thus restrict the clinical applications. Type, size, quantity and refractive index of the fillers into composite exert an influence upon light transmission across the material. Consequently, the light attenuation and the depth of cure may be altered.[1,2] With respect to the organic matrix, the nature of the involved monomer molecules and the degree of conversion obtained in composite resin have an important effect on the mechanical properties,[3] where the higher degrees of cure will improve the final properties of the material.

A higher degree of conversion can be obtained by using a high light intensity.[4] However, this higher intensity may result in greater polymerization shrinkage and greater marginal leakage.[4] Therefore, new photo-activation techniques have been proposed, such as the programmed use of low and high intensities that have shown to be more effective in decreasing the stress generated by polymerization shrinkage, whilst maintaining a high degree of conversion and satisfactory mechanical properties.[5,6] Since the introduction of this method, other photo-activation methods have been suggested, including intermittent light,[7] stepped light, exponential light and, more recently, a new technology employing light-emitting diodes (LEDs).[8] However, these innovative techniques require further investigation before they can be effectively applied in dental practice.

Thus, the aim of this study was to evaluate the depth of cure and knoop hardness of a micro-fine hybrid composite resin, photo-activated using different methods.

## MATERIALS AND METHODS

This study used the Tetric Ceram composite resin (Ivoclar Vivadent), shade A3. Composition and batch no. are reported in Table 1.

**Table 1:** Composition and batch of the Tetric Ceram composite resin

Organic matrix	BisGMA, UDMA, TEGDMA
Batch	F51124 2006-11
Filler Type	Barium glass, ytterbium trifluoride, Ba-Alfluorosilicate glass, silicone dioxide and spheroid mixed oxide
% (Volume)	60
Size (µm)	0.04 - 3.0

The composite resin was placed in square recesses (4 mm long × 4 mm wide × 11 mm deep) of a customized bipartite brass mold and was confined between two opposing acetate strips. A glass slide (1 mm thick) was then placed over this and pressure was applied to accommodate the material. The composite was then irradiated from the top, through the glass slide and acetate strip using different photo-activation methods. Photoactivation was performed with

1. Intermittent method (QTH) (Group I)
2. Continuous method (QTH) (Group II)
3. Exponential method (QTH) (Group III)
4. Continuous method (LED) (Group IV).

Ten specimens were prepared for each photo-activation method.

**Figure 1:**
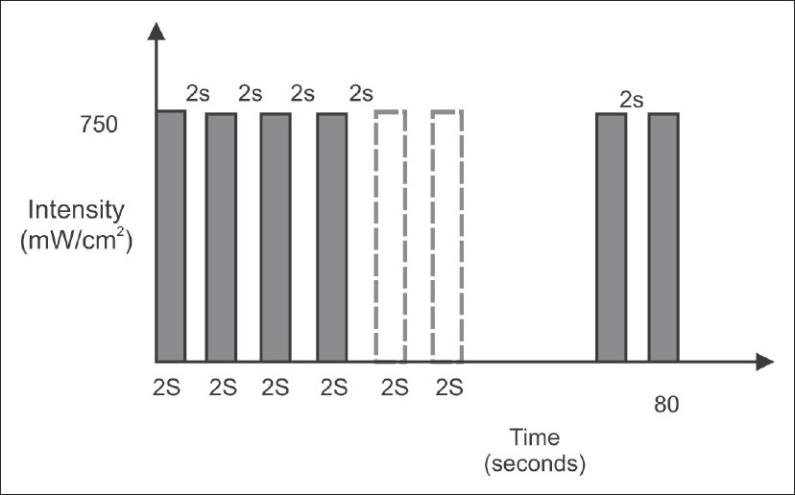
Bar diagram representing the time and intensity of light used in intermittent method (QTH) of photo-activation

For the intermittent method, the curing tip was positioned close to the brass matrix/restorative composite and photo-activation was performed for two seconds, with an intensity of 750 mW/cm2 and two seconds without light, with a total exposure time of 80 seconds, using an Astralis 7 (Ivoclar Vivadent), QTH LCU [Figure 1].

**Figure 2:**
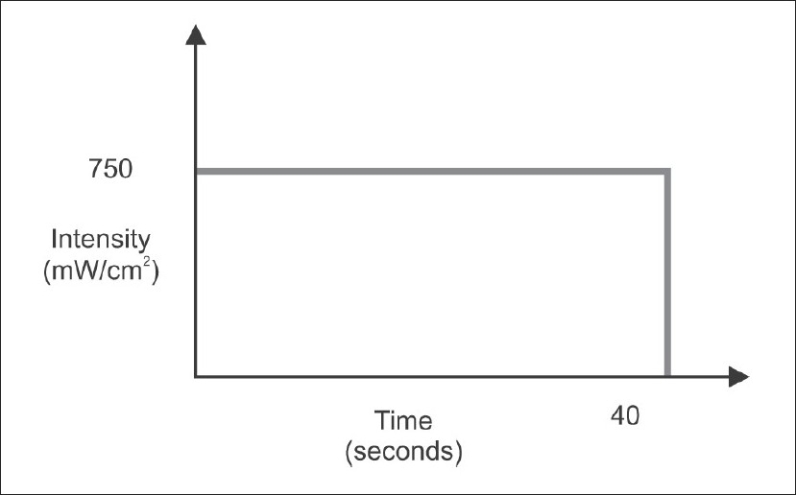
Bar diagram representing the time and intensity of light used in continuous method (QTH) of photo-activation

For the continuous method (QTH), the same curing unit was used and photo-activation was performed for 40 seconds with a high intensity of 750 mW/cm^2^ [Figure 2].

**Figure 3:**
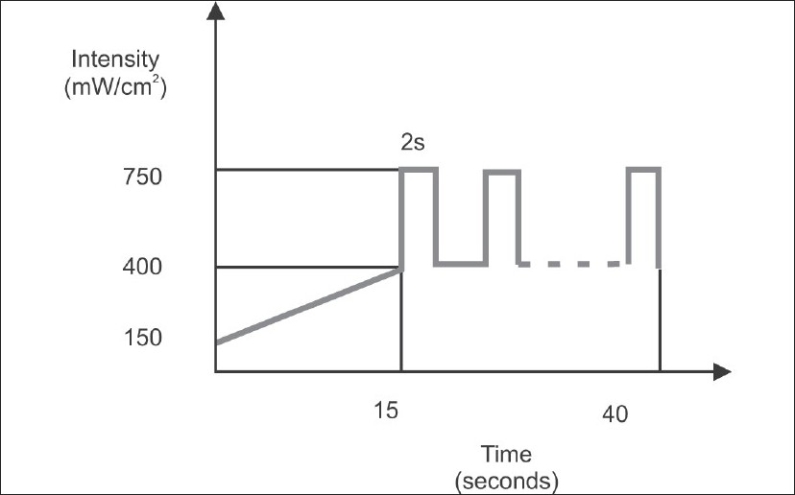
Bar diagram representing the time and intensity of light used in exponential method (QTH) of photo-activation

For the exponential method, the same Astralis 7 (Ivoclar Vivadent), QTH LCU was used; however, the light intensity began initially at 150 mW/cm2 and within 15 seconds it was increased gradually to 400 mW/cm^2^. After that, the intensity was changed between 400 mW/cm2 and 750 mW/cm^2^, every two seconds for 25 seconds, with a total exposure time of 40 seconds [Figure 3].

**Figure 4:**
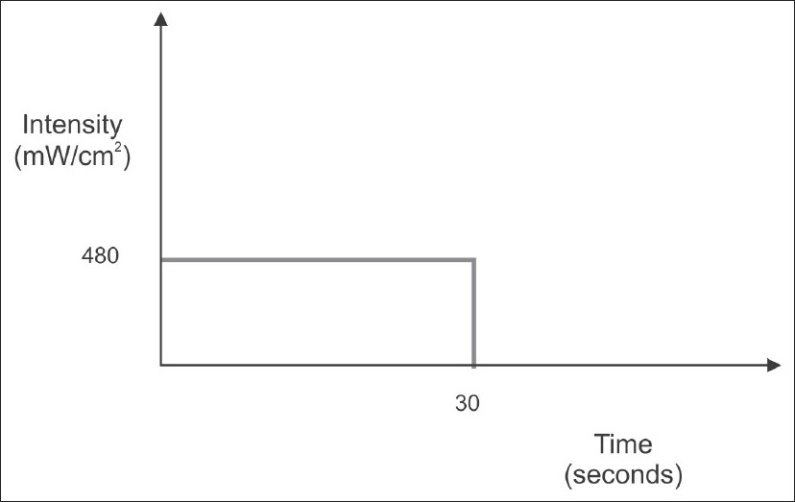
Bar Diagram representing the time and intensity of light used in continuous method (LED) of photo-activation

For the continuous method using LED, BlueLex (Monitex Industrial Co. Ltd) LED LCU was used according to the manufacturer's information. When the battery was fully charged, photo-activation was performed for 30 seconds, with an intensity of 480 mW/cm^2^, using the continuous method of photo-activation [Figure 4]. Photo-activation was performed for 30 seconds; this was recommended by the manufacturer because low intensity LED used for 30 seconds is sufficient to cure the same depth of composite, as can be cured with QTH. The light intensity of both the curing units was measured with a radiometer (Curing Radiometer, Optilux 501) to ensure consistency in the output.

Immediately after photo-activation, the glass slab and acetate strips were removed and the specimens were positioned in the mold to assess knoop hardness and depth of cure.

### A) Depth of cure testing

**Figure 5:**
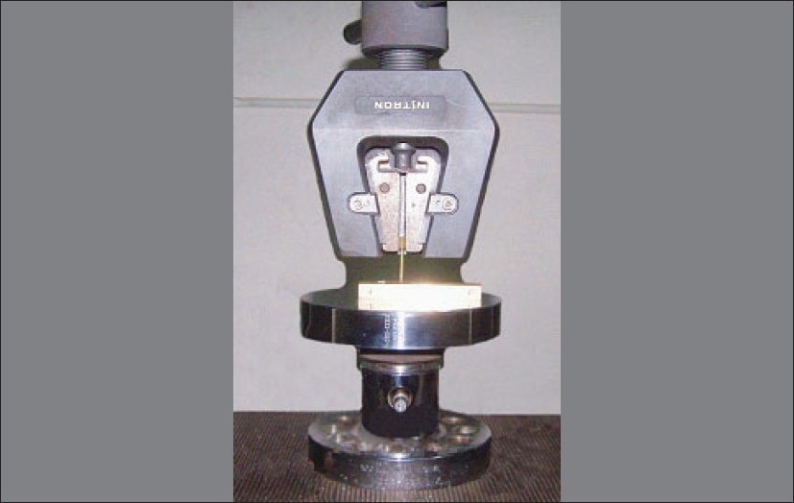
Pictorial illustration of depth of cure by penetration technique

Penetration method: The methodology used in this evaluation was based upon that used by Harrington and Wilson (1993). A microtester (Instron Corporation, Model No. 4206) was used as a penetrometer. Immediately after light curing, the specimens in their molds were inverted, with the unexposed surface facing the penetration needle [Figure 5]. A force of 12.5 N (1250 grams) was exerted after light exposure through a 0.5 mm diameter needle, at the rate of 1 mm/min, in the middle of the unexposed, bottom surface; the depth of penetration was measured digitally.

Depth of cure was measured using the formula:

Depth of cure=Depth of mold (11 mm)-Depth of penetration

### B) Knoop hardness testing

After testing depth of cure, the same cured specimens were subjected to hardness testing using a Digital Microhardness Tester (Matsuzawa Co., Ltd. Model no. MMT-X7 Toshima, Kawabe, Japan). Specimens with light exposed surface were placed centrally below the knoop diamond indenter and a 500 g load was applied through the indenter, with a dwell time of 15 seconds, to assess the knoop hardness number (KHN) of the top surface. The KHN at the top surface was measured at 100× magnification. The KHN corresponding to each indentation was computed by measuring the dimensions of the indentation and using the formula KHN = 14. 2 × (F/d^2^), where F = test load in Newtons; d = longer diagonal of an indentation in millimeter. After determining the top surface KHN, the bipartite brass mold was opened and KHN values of the side surfaces of the composite specimens were measured at 1 mm intervals, from the top surface up to 5 mm depth, using the same testing parameters as described above.

The data was subjected to analysis of variance (anova) and the means were compared by Tukey's test (significance level 5%).

## RESULTS

Table 2 summarizes and compares the depth of cure of different photo-activation methods. The intermittent method (QTH) had the highest depth of cure (6.340 mm), followed by the continuous method (QTH) (5.663mm) and the exponential method (QTH) (5.403mm). The lowest depth of cure was obtained with continuous method (LED) (5.209mm). There was very high statistically significant difference among all the four groups (*P*<0.001).

**Table 2:** Depth of cure according to photo-activation method

Photo-activation method	Means ± Standard Deviation (mm)
Intermittent method (QTH)	6.34 ± 0.093a
Continuous method (QTH)	5.663 ± 0.115b
Exponential method (QTH)	5.403 ± 0.069c
Continuous method (LED)	5.209 ± 0.087d
	

Means followed by different letters are very high statistically different at 5% by Tukey's test.

Table 3 shows that at the surface towards light and up to 1 mm depth, continuous method (LED) demonstrated the highest KHN, whereas exponential method (QTH) showed the lowest KHN. At 2 mm depth, intermittent method (QTH) presented the highest KHN and exponential method (QTH) showed the lowest KHN. At 3 mm, 4 mm and 5 mm, intermittent method (QTH) presented the highest KHN and continuous method (LED) showed the lowest KHN.

**Table 3:** Knoop hardness according to region and photo-activation method

Method	Surface	1 mm	2 mm	3 mm	4 mm	5 mm
Intermittent (QTH)	70.300	70.400	68.300	65.700	59.400	41.800
	(0.675)	(0.843)	(1.252)	(0.823)	(1.647)	(1.229)
Continuous (QTH)	64.400	63.600	62.100	55.600	44.300	30.100
	(0.843)	(0.843)	(0.738)	(0.966)	(1.494)	(1.595)
Exponential (QTH)	62.300	61.300	61.000	53.200	40.300	24.200
	(0.675)	(0.823)	(0.816)	(1.317)	(1.767)	(1.003)
Continuous (LED)	75.200	74.400	65.300	49.900	24.800	9.300
	(0.789)	(0.699)	(1.418)	(0.994)	(0.919)	(1.252)

Means followed by standard deviation (given within parentheses) are very high statistically different at 5% by Tukey's test.

At all the depths, continuous method (QTH) showed higher knoop hardness values, as compared to the exponential method (QTH).

There was very high statistically significant difference in the mean knoop hardness number between all the individual groups at all the depths (*P*<.001), except at 2 mm depth, where the continuous method (QTH) and the exponential method (QTH) showed no statistically significant difference in the mean knoop hardness number (*P*>.05).

## DISCUSSION

The development of new technologies for photoactivation of restorative composite resins has caused great interest among researchers.[1–3,5] However, the real advantages of these techniques are not yet totally known. Before these methods can be clinically applied, the final properties of photo-activated composites must be evaluated.

The results of this study showed that depth of cure is strongly affected by photo-activation methods. The intermittent, continuous and exponential light methods supply energy for photo-activation via halogen lamps, and white light must be filtered to emit only the blue spectrum of the visible light. To generate blue light, the lamps must be heated to very high temperatures,[4] resulting in emission of heat through the curing light tip.[5,7] This heat transmission to the material may be, in part, responsible for the higher depth of cure values achieved using these methods, because the heat may increase the mobility of the monomers, increasing the probability of occurrence of conversion.

Another factor that may have caused the difference between intermittent method and the continuous and exponential methods is the total amount of energy supplied to the composite for polymerization. According to Sakaguchi and Berge,[9] maximum light intensity is achieved at 0.55 s and then it decreases, signifying that even with continuous method (750 mW/cm^2^), the amount of energy supplied is not constant. Conversely, the intermittent method employs 2 s of light exposure followed by 2 s without light; that is, maximum light intensity peak is achieved every time the light is emitted. Since the polymerization process seems more dependent on the total energy available for photo-activation than the light intensity property,[10] this method may provide a higher amount of energy to the material, which may explain the higher depth of cure values achieved using the intermittent method.

Light-emitting diodes or LED, the more recent technology developed for photo-activation of resinous materials, combines two different semiconductors (p - n junctions). When a voltage is applied, the ‘electrons’ and ‘holes’ recombine at the LED's p - n junctions leading, in the case of gallium nitride LEDs, to emit of blue light. The spectral output of gallium nitride blue LED falls conveniently within the absorption spectrum of the camphoroquinone photo-initiator (400-500 nm) presented in most light-activated composite resins. Therefore, no filters are required in LED LCU's.[11,12] However, LED demonstrated the lowest depth of cure. This result may be due to the comparatively low light intensity (480 mW/cm^2^), which provides a very short time duration for the light to reach the deeper regions of the material, and due to the absence of heat emission.

According to Peutzfeldt *et al.*,[13,14] when light curing units are studied, an important parameter is the amount of light energy of appropriate wavelength emitted during irradiation. This energy is calculated as the product of the output of the curing light unit and the time of irradiation; it may be called energy density. This could explain the lower depth of cure obtained with the LED method, as compared to the methods that use the halogen lamp.

The knoop hardness test showed that up to a depth of 1 mm, LED showed the highest KHN. This is because although the LED light in this study was applied for a shorter time than the QTH light, it was more effective than other photo-activation methods using QTH curing unit in polymerizing the top surface and up to a depth of 1 mm. This finding is consistent with the findings of previous investigations.[15] Halogen curing units use filters to eliminate all wavelengths, except blue light. Therefore, some of the emitted photons might be out of the spectrum of absorption of camphoroquinone and the triplex state of camphoroquinone might not be fully activated.

At 2 mm depth, the intermittent method presented the highest KHN and continuous method using LED showed the second highest KHN. This is again due to higher energy density, heat generated and longer duration of exposure employed during the intermittent method. At a depth of 2 mm, continuous and exponential methods using QTH showed intermediate values and there was no significant difference between them. This result demonstrates that despite the particular characteristics of each method, the light intensity and exposure time were enough to adequately polymerize this thickness of composite.[16]

At a depth of 3mm, the continuous method using LED demonstrated the lowest hardness value. The lower value observed with LED may be due to the fact that the light is absorbed and/or scattered when the thickness increases,[18,19] consequently decreasing the amount of energy for photo-activation. Also, low intensity and reduced exposure time compared to QTH units may be an added factor. A reduced irradiation time is claimed to be satisfactory by light manufacturers, due to the high radiation intensity of the units. However, studies have shown that an exposure time of 40 seconds is required to provide composites with uniform and high KHN, when LED units are employed. Despite this scattering and absorbance of light, all other methods supplied higher amounts of energy to the composite, and, thus, provided higher hardness values at 3 mm depth. This is due to the fact that longer wavelengths of the QTH curing unit penetrated deep into the composite, as compared to the shorter wavelengths of the LED curing unit.

At a depth of 4 mm and 5 mm, the intermittent method demonstrated the highest KHN. LED presented the lowest hardness, with statistically significant difference from other methods. The continuous and exponential methods showed intermediate KHN values. This result may be due to the total amount of energy supplied to the camphoroquinone, even at great depth. The total energy is related to the exposure time and light intensity generated by each method, i.e., the energy density. The intermittent method was able to provide a higher amount of energy at these depths, due to the intermittence itself, where the maximum intensity is achieved at 0.55 seconds; it then decreases.[20] The continuous method (QTH) showed higher KHN than the exponential method at almost all the depths. This could be due to the fact that in the exponential method, initially using the gradually increasing low intensity, it appeared to minimize the compensatory effects of high light intensities. Also, polymerization of resin associated with the initial cure may be sufficient to interfere with light transmission and severely decrease the amount of light reaching the bottom surface of composite restorations.[20]

## CONCLUSION

It was concluded that all the photo-activation methods provided depth of cure values that fulfilled the requirements of ISO 4049. The intermittent method using QTH showed the highest depth of cure while the continuous method using LED demonstrated the lowest depth of cure, thereby proving that the latter is less effective for deep restorations when using a micro-fine hybrid composite resin.

Therefore, for composite restorations, the use of intermittent method for photo-activation, with high intensity curing light to improve the polymerization in the bottom surface of the first increments of micro-fine hybrid composite resin is preferred.
